# A Comprehensive Review of Multimodal Medical Image Fusion: Techniques, Evaluation, and Future Directions

**DOI:** 10.3390/s26165067

**Published:** 2026-08-10

**Authors:** Pengquan Han, Cuiyin Liu, Cuiwei Wang

**Affiliations:** Faculty of Information Engineering and Automation, Kunming University of Science and Technology, Kunming 650500, China; hanpengquan@stu.kust.edu.cn (P.H.); liucuiyin@kust.edu.cn (C.L.)

**Keywords:** multimodal medical image fusion, medical imaging modalities, image fusion methods, deep learning, Mamba, quality assessment metrics

## Abstract

In recent years, multimodal medical image fusion (MMIF) has attracted significant attention due to its ability to integrate complementary information from different medical imaging modalities and provide more comprehensive information for clinical analysis. By combining anatomical information from modalities such as computed tomography (CT) and magnetic resonance imaging (MRI) with functional information from positron emission tomography (PET) and single-photon emission computed tomography (SPECT), MMIF can improve image quality and support subsequent tasks such as disease analysis, lesion detection, image segmentation, and treatment planning. This review provides a comprehensive overview of MMIF from theoretical and technical perspectives. First, commonly used medical imaging modalities and publicly available medical image databases are summarized and compared. Subsequently, the general workflow, fusion levels, and quality requirements of MMIF are introduced. Representative fusion techniques are then systematically reviewed, including spatial-domain methods, transform-domain methods, sparse representation-based methods, deep learning-based methods, hybrid methods, and emerging Mamba-based approaches. In addition, commonly used image fusion quality assessment metrics are analyzed, and the reported quantitative performance of representative MMIF methods is compared and discussed. Finally, current challenges and future development trends of MMIF are presented, including robustness, clinical translation, and emerging multimodal learning paradigms.

## 1. Introduction

As an important research topic in computer vision and image analysis, image fusion has attracted considerable attention due to its broad applicability in fields such as virtual and augmented reality, environmental monitoring, satellite surveillance, remote sensing [1], infrared imaging [2], and clinical diagnosis [3]. By effectively integrating complementary information from multiple imaging sources, image fusion can significantly improve the perception and identification of critical targets and pathological structures that are difficult to characterize using a single modality, including malignant tumors, lesion regions, and cancer cells.

With the rapid advancement of medical imaging technologies, a variety of imaging modalities have been developed and extensively employed in clinical practice. However, a single imaging modality is generally incapable of simultaneously providing comprehensive anatomical and functional information required for accurate diagnosis. Furthermore, each modality possesses inherent strengths and limitations in terms of spatial resolution, contrast, radiation exposure, acquisition cost, and imaging efficiency. Consequently, different modalities offer complementary information regarding the same anatomical structure or pathological region. To effectively exploit such complementary characteristics, multimodal medical image fusion has emerged as a crucial technique for integrating heterogeneous information from multiple imaging modalities, thereby improving disease monitoring, lesion characterization, and clinical diagnosis [4].

A variety of medical imaging modalities, including Magnetic Resonance Imaging (MRI), Computed Tomography (CT), Positron Emission Tomography (PET), Single-Photon Emission Computed Tomography (SPECT), X-ray imaging, and Ultrasound (US), are routinely utilized in clinical diagnosis. Structural imaging modalities such as MRI, CT, X-ray, and US primarily provide high-resolution anatomical information, enabling the visualization of lesion location, size, morphology, and structural alterations in surrounding tissues. In contrast, functional imaging modalities, including PET, functional MRI (fMRI), and SPECT, are capable of reflecting metabolic activity, physiological processes, and tissue functionality, thereby offering valuable insights into tumor progression and biological behavior. Since these modalities provide complementary anatomical and functional information, Multimodal Medical Image Fusion (MMIF) has become an important research topic in medical image analysis. By integrating two or more geometrically registered images acquired from different modalities into a unified representation, MMIF aims to generate a fused image that preserves salient information from each source image while enhancing visual quality and diagnostic value, thereby supporting more accurate clinical diagnosis and treatment planning [3,5,6].

The integration of multimodal medical images enables the complementary fusion of anatomical and functional information obtained from different imaging modalities, such as CT, MRI, PET, and SPECT. By leveraging the strengths of each modality, MMIF can enhance image contrast, structural representation, and visual perception, thereby improving the detection of subtle pathological abnormalities, including tumors and vascular lesions. Moreover, the fusion of heterogeneous information from different imaging perspectives facilitates more accurate lesion localization and characterization, which plays a critical role in treatment planning and therapeutic assessment. As a result, clinicians can obtain more comprehensive diagnostic information from a single fused image, significantly improving both diagnostic accuracy and clinical workflow efficiency.

**Figure 1:**
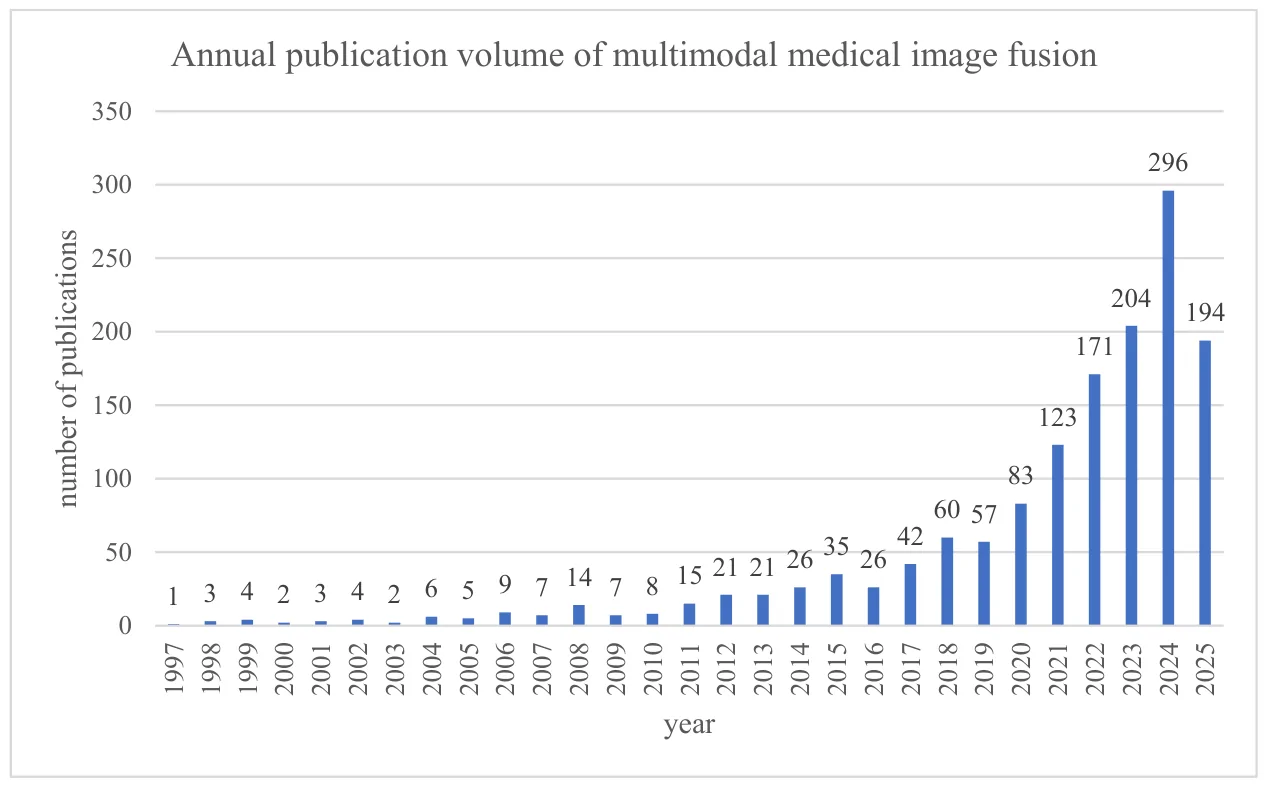
Annual publication output in the field of multimodal medical image fusion (MMIF) from 1997 to Q2 2025, based on records retrieved from the Web of Science (WoS) database.

Beyond its clinical benefits, MMIF has become an increasingly active research area in medical image analysis. By enabling the effective integration of heterogeneous information from multiple imaging modalities, MMIF supports more accurate diagnosis, treatment planning, and longitudinal disease assessment. The growing importance of this field is reflected in the rapidly increasing volume of related publications. As shown in Figure 1, the annual number of MMIF publications from 1997 to the second quarter of 2025 exhibits a clear upward trend, based on bibliometric records retrieved from the Web of Science (WoS) database.

The rapid increase in MMIF-related publications can be largely attributed to three factors. The continuous expansion of medical image repositories has provided abundant datasets for algorithm development and validation. In addition, advances in image processing, signal analysis, and artificial intelligence have significantly accelerated methodological innovation. Furthermore, the growing demand for comprehensive diagnostic information in computer-aided clinical applications has further stimulated the development and adoption of multimodal image fusion techniques. As the field has evolved, a substantial number of review and survey articles have been published to systematically summarize the progress, challenges, and future directions of medical image fusion research [7].

Numerous survey and review articles have comprehensively summarized the development of MMIF techniques from different perspectives. Existing reviews mainly focus on fusion methodologies, imaging modalities, fusion frameworks, image registration, quality assessment metrics, and clinical applications.

For example, James et al. [8] discussed several open challenges in MMIF research, including limited algorithmic innovation, inaccurate image registration, and unresolved issues in feature extraction and processing. Their review analyzed MMIF from the perspectives of fusion techniques, imaging modalities, and organ-specific applications. Du et al. [3] systematically summarized the complete MMIF pipeline, including image decomposition, fusion, reconstruction, fusion rules, and quality evaluation, while comparing several representative fusion approaches using multiple assessment metrics. Zhang et al. [9] reviewed the principles and development trends of feature-level medical image fusion and discussed the application of different fusion strategies in medical imaging.

In addition, image registration, which serves as a prerequisite for accurate image fusion, has also been extensively investigated. El-Gamal et al. [10] analyzed the classification of medical image registration methods and discussed the potential clinical applications and future development trends of medical image fusion. Meher et al. [11] provided one of the earliest comprehensive classifications of region-based image fusion methods and summarized objective fusion quality evaluation metrics. Although their review covered a broad range of fusion tasks, including multimodal medical image fusion, infrared-visible image fusion, multi-focus fusion, and multi-exposure fusion, it did not specifically focus on medical image fusion.

Furthermore, Hermessi et al. [12] categorized MMIF methods into pixel-level, feature-level, and decision-level fusion according to the level of information abstraction, and reviewed seven representative fusion strategies. Azam et al. [13] presented a comprehensive overview of medical imaging principles, fusion methodologies, multimodal medical image databases, and disease-oriented applications. More recently, Khan et al. [14] summarized multimodal imaging databases, imaging modalities, and fusion procedures, while highlighting the application of deep learning techniques in COVID-19 detection systems.

Unlike previous review articles that mainly focus on specific aspects of MMIF, such as fusion algorithms, imaging modalities, databases, or evaluation metrics, this review provides an integrated analysis of the MMIF research landscape from technical, methodological, and clinical perspectives. Specifically, this work provides a structured framework covering medical imaging modalities, publicly available databases, fusion workflows, methodological classification and evolution, evaluation strategies, and practical challenges. Beyond summarizing existing approaches, representative MMIF methods are comparatively analyzed according to a set of unified criteria, including information representation capability, computational efficiency, data requirements, generalization ability, robustness, interpretability, and clinical applicability. The main objectives of this study are threefold: (1) to systematically categorize existing MMIF techniques and their applications; (2) to critically analyze the limitations and challenges affecting practical deployment, including evaluation validity, robustness, and clinical translation; and (3) to discuss emerging trends and future research directions in multimodal medical image fusion.

This review aims to provide researchers with a comprehensive understanding of the evolution, current status, and future opportunities of MMIF, while offering practical guidance for selecting appropriate fusion strategies under different application scenarios.

The remainder of this paper is organized as follows. Section 2 reviews the classification of medical imaging modalities. Section 3 introduces six publicly available multimodal medical imaging databases and discusses their characteristics and limitations. Section 4 describes the general workflow of medical image fusion. Section 5 presents a systematic classification, comparative analysis, and discussion of the limitations of existing multimodal image fusion techniques. Section 6 summarizes commonly used fusion quality assessment metrics and discusses their limitations in clinical validation. Section 7 discusses open challenges and future research directions. Finally, Section 8 concludes the paper.

## 2. Medical Imaging Modalities

Medical imaging aims to noninvasively visualize the internal structures, tissues, and physiological processes of the human body, thereby providing critical information for clinical diagnosis and treatment. Different imaging modalities are based on distinct physical principles and acquisition mechanisms, resulting in complementary anatomical, functional, and metabolic information [13].

According to their underlying physical imaging mechanisms, the medical imaging modalities involved in MMIF can generally be categorized into two groups: acoustic energy-based imaging and electromagnetic energy-based imaging. Acoustic imaging primarily refers to Ultrasound (US), which generates real-time dynamic images by exploiting the propagation, reflection, and scattering characteristics of ultrasonic waves in biological tissues. Electromagnetic imaging modalities acquire diagnostic information through various interactions between electromagnetic signals and biological tissues. Representative modalities include Magnetic Resonance Imaging (MRI), Computed Tomography (CT), Positron Emission Tomography (PET), Single-Photon Emission Computed Tomography (SPECT), and X-ray imaging. Owing to differences in operating frequencies, signal acquisition mechanisms, and imaging principles, these modalities provide complementary anatomical, functional, and metabolic information [15].

**Figure 2:**
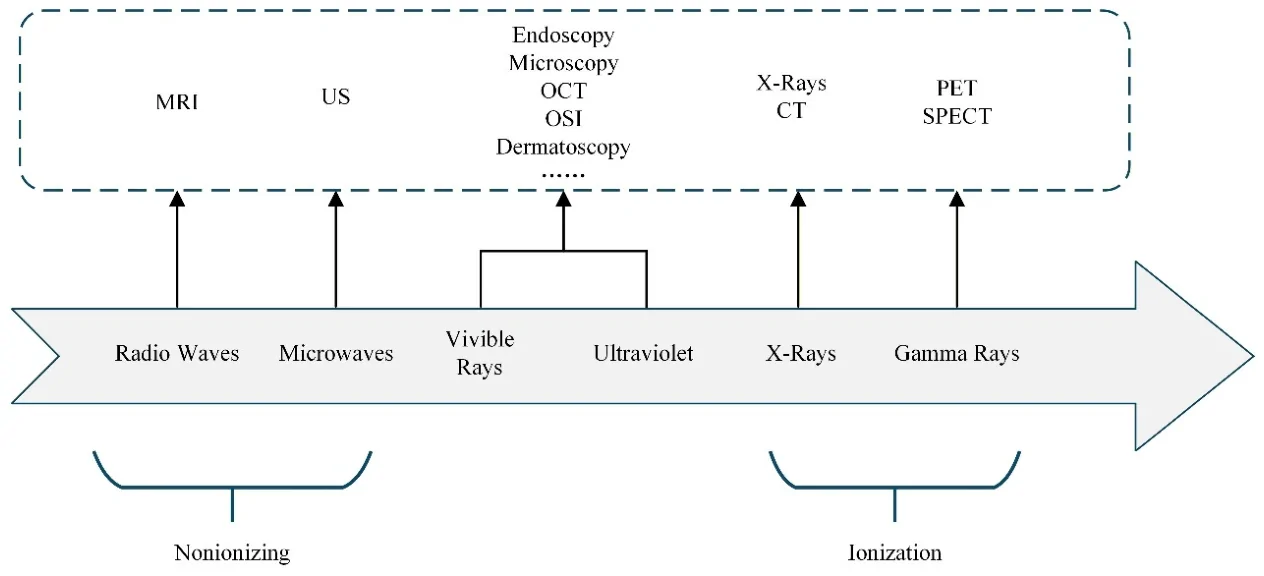
Distribution of major medical imaging modalities within the electromagnetic spectrum.

As illustrated in Figure 2, major medical imaging modalities occupy different regions of the electromagnetic spectrum and generate medical images using different forms of energy. From the perspective of radiation characteristics, X-ray imaging, CT, PET, and SPECT are classified as ionizing imaging modalities, whereas MRI and US are categorized as non-ionizing imaging modalities because they do not expose patients to ionizing radiation during image acquisition [16].

**Figure 3:**
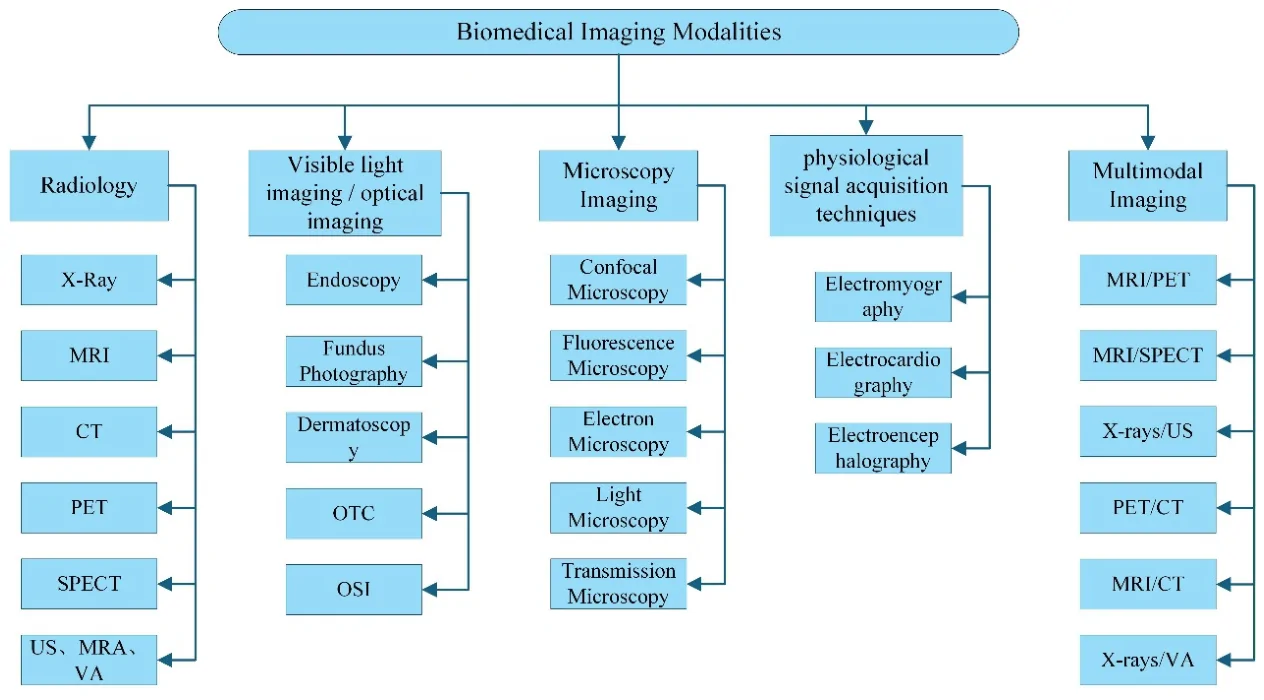
Taxonomy of major biomedical imaging and physiological signal modalities.

In addition to the aforementioned clinical imaging modalities, biomedical imaging encompasses several other important branches, including microscopic imaging, optical imaging, and physiological signal acquisition. Microscopic imaging techniques, such as Light Microscopy, Confocal Microscopy, Fluorescence Microscopy, and Electron Microscopy, are widely used for the observation of cellular and tissue microstructures. Optical imaging modalities include Optical Coherence Tomography (OCT), Optical Scatter Imaging (OSI), Endoscopy, Dermatoscopy, and Fundus Photography, which provide structural and morphological information about superficial tissues and organs. Furthermore, physiological signal acquisition techniques, including Electrocardiography (ECG), Electromyography (EMG), and Electroencephalography (EEG), are extensively employed to record the electrical activities of biological tissues and organs. A taxonomy of the major biomedical imaging and physiological signal modalities discussed in this section is illustrated in Figure 3.

Although different imaging modalities provide complementary anatomical, functional, and metabolic information, the quality of the acquired images is strongly influenced by acquisition hardware, sensing mechanisms, and imaging protocols. These acquisition-related factors have an important impact on subsequent image registration and fusion, as discussed in Section 4.

Medical imaging modalities can also be classified according to whether the acquisition process requires access to the interior of the body, namely invasive imaging and non-invasive imaging. Invasive imaging techniques typically involve surgical incisions, needle punctures, catheter insertions, biopsies, or other interventional procedures to introduce medical instruments into the body for obtaining diagnostic information from specific organs or tissues. In contrast, non-invasive imaging techniques acquire anatomical, functional, or pathological information without disrupting tissue integrity or inserting instruments into the body. Common clinical imaging modalities, including MRI, CT, PET, SPECT, X-ray imaging, and US, are generally classified as non-invasive imaging techniques. Table 1 summarizes the major medical imaging modalities involved in MMIF and their corresponding classifications.

It is worth noting that the concept of multimodality in biomedical applications has gradually extended beyond conventional image-based fusion. In addition to integrating different medical imaging modalities, emerging multimodal biomedical systems increasingly combine imaging data with physiological signals, bioelectrical measurements, wearable sensors, and other non-invasive sensing technologies. For example, electrocardiography (ECG), electroencephalography (EEG), electromyography (EMG), and electrical impedance tomography (EIT) provide complementary information regarding physiological activities and tissue characteristics. By integrating heterogeneous biomedical information with advanced computational models and artificial intelligence techniques, these systems enable more comprehensive physiological monitoring and intelligent analysis. Therefore, MMIF can be regarded as an important component of a broader multimodal biomedical intelligence framework.

## 3. Multimodal Medical Imaging Databases

In this section, several representative multimodal medical imaging databases are introduced. In MMIF studies, the source images generally require accurate registration and alignment before the fusion process to ensure the consistency of anatomical structures and functional information. Therefore, publicly available and freely accessible databases have become important resources for the development, validation, and performance evaluation of fusion algorithms.

Brain imaging has been the dominant application area in MMIF research due to the availability of diverse imaging modalities, established registration techniques, and several publicly accessible datasets combining anatomical and functional information, such as MRI with PET or SPECT. However, the application of MMIF is not limited to neuroradiology. Recent studies have extended multimodal fusion techniques to other clinical fields, including oncology, cardiology, pulmonary imaging, musculoskeletal imaging, and surgical navigation, where complementary information from different imaging modalities can support diagnosis, treatment planning, and intervention guidance. Therefore, although brain imaging represents a major research focus, MMIF is a broader interdisciplinary technology with increasing applications across multiple medical domains.

It should be noted that publicly available MMIF datasets are currently concentrated in neuroimaging, which partly explains the predominance of brain-related studies in this field. Expanding publicly accessible datasets covering other organs and clinical scenarios remains important for improving the generalizability of MMIF methods.

This review focuses on six publicly available multimodal medical imaging databases, namely OASIS, AANLIB, TCIA, ADNI, MIDAS, and OCTA-500. These databases contain medical images of various organs affected by different diseases and provide multimodal imaging data acquired from either the same patient or different subjects. Such datasets facilitate the objective evaluation and comparison of MMIF algorithms under diverse imaging conditions. A comprehensive comparison of these databases is provided in Table 2 at the end of this section.

### 3.1. OASIS Database

The OASIS database is a publicly available neuroimaging dataset designed to support research on brain structure, aging, and neurological disorders. It provides cross-sectional and longitudinal MRI data collected from healthy individuals as well as patients with cognitive impairment and neurodegenerative diseases. The OASIS database comprises three publicly available neuroimaging datasets, namely OASIS-1, OASIS-2, and OASIS-3, which are widely used for brain imaging research and analysis. OASIS-1, released in 2007, contains cross-sectional MRI data from 416 subjects aged 18 to 96 years, comprising a total of 434 imaging sessions [17]. OASIS-2, released in 2009, provides longitudinal MRI data from 150 subjects aged between 60 and 96 years, totaling 373 imaging sessions. OASIS-3, released in 2019, significantly expanded the scale of the database by incorporating retrospective data from more than 1000 participants. In addition to structural MRI data, OASIS-3 also provides PET imaging acquired using three different radiotracers, enabling multimodal investigations of brain structure, function, and disease progression.

### 3.2. AANLIB Database

The Whole Brain Atlas, provided by Harvard Medical School, is a publicly accessible online brain imaging resource that has been widely used in neuroimaging research and multimodal image fusion studies [18,19]. The database contains both normal and pathological brain images. Normal brain images are available in two-dimensional (2D) and three-dimensional (3D) formats, whereas pathological cases are categorized into several disease groups, including stroke, brain tumors, degenerative disorders, and infectious diseases. The images are stored in GIF format, facilitating convenient access and visualization.

The Whole Brain Atlas focuses exclusively on brain imaging and includes multiple imaging modalities, such as MRI, CT, PET, and SPECT. For MRI data, both T1-weighted and T2-weighted sequences are provided. In addition, users can directly view fused PET–MRI images through the online platform. Since multimodal images from the same case are generally provided in a spatially aligned form, the database has become one of the most widely used public datasets for brain-oriented MMIF research.

### 3.3. TCIA Database

The TCIA (The Cancer Imaging Archive) is one of the largest publicly available repositories of medical imaging data for cancer research. The database primarily stores imaging data in the Digital Imaging and Communications in Medicine (DICOM) format and contains datasets acquired from a wide range of imaging modalities and anatomical sites.

TCIA includes imaging data from multiple modalities, such as CT, MRI, PET, Mammography (MG), and other specialized imaging techniques. In addition to imaging data, the archive provides a variety of complementary resources, including clinical outcomes, treatment information, genomic data, pathology reports, and expert annotations. These datasets cover numerous cancer types and anatomical regions, including the breast, chest, brain, colon, and many other organs. Owing to its large scale, multimodal nature, and rich clinical metadata, TCIA has become one of the most widely used public databases for cancer imaging research, image analysis, machine learning, and multimodal medical image fusion studies.

### 3.4. ADNI Database

The ADNI (Alzheimer’s Disease Neuroimaging Initiative) was established to investigate the progression of Alzheimer’s disease through the integration of neuroimaging, biomarkers, and clinical assessments. Its primary objective is to monitor structural and functional changes in the brain associated with normal aging, mild cognitive impairment (MCI), and Alzheimer’s disease (AD).

ADNI collects a wide range of data from volunteer participants, including neuroimaging data, cognitive assessments, genetic information, and biological markers. These data are freely available to qualified investigators upon application and approval. Among the available imaging modalities, Magnetic Resonance Imaging (MRI) and Positron Emission Tomography (PET) constitute the core imaging resources of the database and have been extensively utilized in studies of neurodegenerative diseases, disease progression analysis, and multimodal medical image fusion.

### 3.5. MIDAS Database

The MIDAS (Michigan Image Database and Analysis System), developed by the University of Michigan, is a medical imaging repository that provides multimodal biomedical imaging datasets for medical image analysis and computer-aided diagnosis research [20]. It supports multiple medical imaging formats, with DICOM as the primary standard, and contains heterogeneous datasets acquired from various imaging modalities and clinical applications.

Among the datasets hosted by MIDAS, the Retrospective Image Registration Evaluation (RIRE) project is one of the most widely used benchmarks for multimodal image registration and analysis. The RIRE dataset consists of registered CT, MRI, and PET images and has been extensively adopted for multimodal image registration, image fusion, and other medical image processing tasks.

### 3.6. OCTA-500 Database

The OCTA-500 dataset, released by Qiang Chen and colleagues at Nanjing University of Science and Technology, is one of the largest publicly available Optical Coherence Tomography Angiography (OCTA) datasets. It contains OCTA scans from 500 subjects acquired under two different fields of view (FOVs), together with corresponding Optical Coherence Tomography (OCT) volumes for multimodal analysis. In addition, the dataset provides six projection images, four categories of clinical annotations (age, gender, eye laterality, and disease status), and seven segmentation labels, including large vessels, capillaries, arteries, veins, 2D and 3D foveal avascular zones (FAZ), and retinal layers [21].

The comprehensive multimodal data and annotations make OCTA-500 a widely used benchmark for retinal image segmentation, disease diagnosis, multimodal image fusion, and retinal image analysis, providing valuable structural and vascular information for ophthalmic research [21].

Although public multimodal medical imaging databases have greatly promoted MMIF research, several limitations remain. Existing datasets differ in sample size, modality diversity, clinical variability, and annotation availability. Limited subjects, specific acquisition protocols, and insufficient clinical annotations may restrict the evaluation of model robustness, generalizability, and clinical applicability. Moreover, demographic biases and limited population diversity may further affect model transferability to broader clinical scenarios. Therefore, future MMIF research requires larger, more diverse, and clinically representative datasets, together with multi-center validation, to reduce dataset bias and bridge the gap between benchmark evaluation and real-world deployment.

## 4. Medical Image Fusion Procedure

In general, the medical image fusion process consists of five major stages: multimodal image acquisition, image preprocessing, image registration, image fusion, and performance evaluation.

The first stage is multimodal image acquisition, which provides the source data for subsequent fusion. The quality of acquisition directly affects fusion performance, as missing information cannot be recovered by subsequent algorithms. Different imaging systems employ distinct sensing mechanisms, detector configurations, and acquisition protocols, resulting in variations in spatial resolution, contrast, and noise characteristics. Acquisition-related factors, including hardware calibration, patient motion, electronic noise, and scanning parameters, may introduce artifacts that propagate through preprocessing, registration, and fusion. Therefore, high-quality acquisition and optimized system design are essential for reliable multimodal image fusion. Recent studies on system-level optimization, such as electrode excitation strategies in three-dimensional electrical impedance tomography (3D EIT), further demonstrate the importance of integrating hardware design with image processing algorithms.

During the preprocessing stage, the primary objective is to reduce or eliminate noise and artifacts from source images to improve image quality and facilitate subsequent processing tasks [22]. Noise reduction is particularly important because image noise may adversely affect feature extraction and degrade fusion performance. Common preprocessing operations include denoising, contrast enhancement, normalization, and artifact correction.

Following preprocessing, image registration is performed to establish spatial correspondence among multimodal images. A typical image registration framework consists of four key steps: (1) feature extraction, (2) feature matching, (3) transformation parameter estimation, and (4) image resampling and geometric transformation [23]. In this process, one image is selected as the reference image, while the remaining images are geometrically transformed to achieve spatial alignment with the reference image [10]. Registration plays a crucial role in medical image fusion by compensating for differences caused by image acquisition, patient movement, rotation, scaling, and geometric distortions, thereby ensuring the accuracy of subsequent fusion operations.

After preprocessing and registration, image fusion techniques are applied to integrate complementary information from multiple source images. Generally, image decomposition algorithms are first employed to decompose the source images into multiple sub-bands or feature components. Subsequently, fusion rules are designed to combine salient information extracted from different modalities. Finally, image reconstruction algorithms are used to generate the fused image from the fused feature representations.

**Figure 4:**
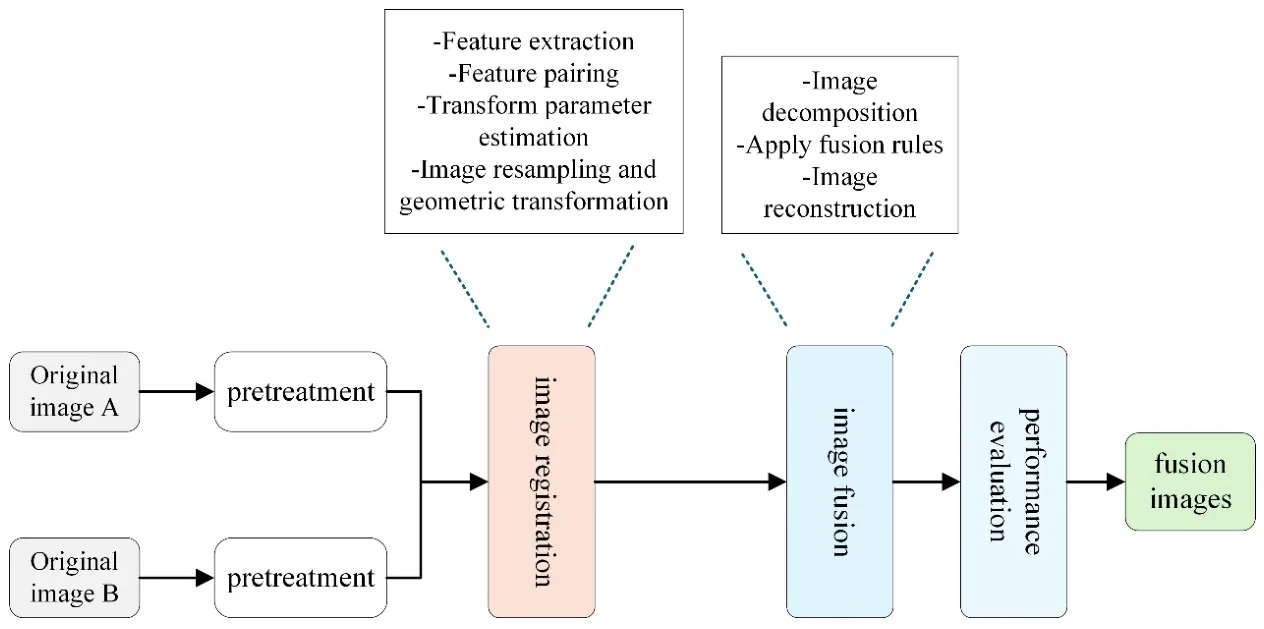
General framework of multimodal medical image fusion.

The final stage involves fusion quality assessment, which evaluates the effectiveness of the fusion process and the quality of the resulting image. Performance evaluation methods can generally be categorized into subjective assessment and objective assessment. Subjective evaluation relies on human visual perception, whereas objective evaluation employs quantitative metrics to measure information preservation, structural similarity, visual fidelity, and other characteristics of the fused image. The overall workflow of medical image fusion is illustrated in Figure 4.

Multimodal Medical Image Fusion (MMIF) is the process of integrating multiple images acquired from one or more imaging modalities into a single composite image, with the objective of improving image quality, enhancing diagnostic accuracy, and preserving complementary information contained in the source images [24]. Medical image fusion commonly involves modalities such as MRI, CT, PET, and SPECT [8]. Among these modalities, PET and SPECT provide functional and metabolic information, such as tissue metabolism, blood perfusion, and physiological activity, but suffer from relatively low spatial resolution. In contrast, MRI, US, and CT offer high-resolution anatomical and structural information but provide limited functional information. Consequently, multimodal medical image fusion typically combines functional and structural images to generate a more comprehensive representation of the underlying anatomy and physiology, thereby facilitating more accurate clinical diagnosis and decision-making.

In MMIF research, investigators first identify the anatomical region or organ of interest. Subsequently, two or more imaging modalities are selected according to the clinical objective, and appropriate fusion techniques are employed to integrate the complementary information contained in the source images. To quantitatively evaluate the effectiveness of fusion algorithms, a series of performance evaluation metrics are generally adopted [25]. Compared with the original source images, the fused image is expected to provide more comprehensive and informative representations of the scanned anatomical region.

**Figure 5:**
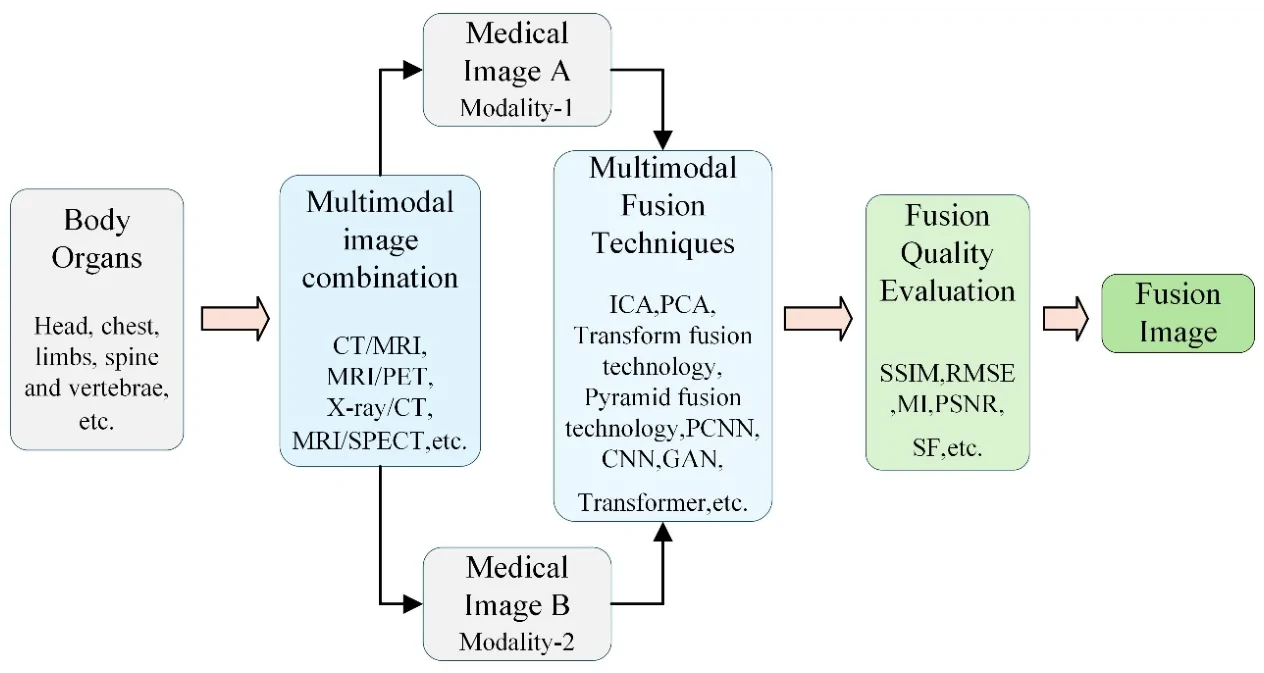
General workflow of multimodal medical image fusion.

An ideal fusion image should satisfy several requirements: (a) it should preserve as much complementary and detailed information from the source images as possible; (b) the fusion process should not introduce artificial information or visual artifacts that may mislead clinicians or adversely affect subsequent image analysis tasks; and (c) it should minimize image degradation caused by noise, distortion, or registration errors [8,26]. The overall workflow of multimodal medical image fusion is illustrated in Figure 5.

Beyond conventional image-to-image fusion, the concept of multimodality in biomedical applications has recently expanded toward the integration of heterogeneous biomedical information. In addition to combining different imaging modalities, such as MRI, CT, PET, and SPECT, emerging multimodal biomedical systems increasingly incorporate physiological signals, wearable sensors, and computational models to achieve more comprehensive health monitoring and intelligent decision-making. These systems often combine heterogeneous data sources with advanced artificial intelligence techniques, including deep neural networks and physics-based modeling approaches such as finite element methods (FEM). Unlike conventional MMIF, which mainly focuses on enhancing visual representation by integrating complementary image information, multimodal biomedical data fusion aims to exploit correlations among different sensing domains. These systems often combine heterogeneous data sources with advanced artificial intelligence techniques, including deep neural networks and physics-based modeling approaches such as finite element methods (FEM), to learn cross-modal representations and achieve intelligent data integration.

## 5. Multimodal Medical Image Fusion Techniques

Multimodal medical image fusion generally begins with the preprocessing of source images, which typically includes noise reduction, contrast enhancement, and intensity correction to improve image quality and ensure the reliability of subsequent processing stages. Subsequently, image registration techniques are employed to establish accurate spatial correspondence among images acquired from different imaging modalities, thereby enabling effective integration of complementary information.

Following registration, feature extraction is performed to obtain representative information from each modality. These features may include texture patterns, edge structures, intensity distributions, and spectral characteristics. The selection of features largely depends on the inherent properties of the imaging modalities and their significance in specific clinical applications. Finally, an appropriate fusion strategy is adopted according to the characteristics of the source images and the requirements of the target application. The extracted information from different modalities is then combined to generate a fused image that contains more comprehensive anatomical and functional information than any individual source image.

### 5.1. Classification of MMIF Methods

Over the past decades, MMIF methods have evolved into three major categories: traditional, deep learning-based, and hybrid methods. Traditional approaches mainly include spatial-domain, transform-domain, sparse representation-based, and other model-driven methods. Deep learning-based methods can be divided into end-to-end and non-end-to-end frameworks, while hybrid methods combine traditional techniques with deep learning models to exploit the strengths of both [27].

**Figure 6:**
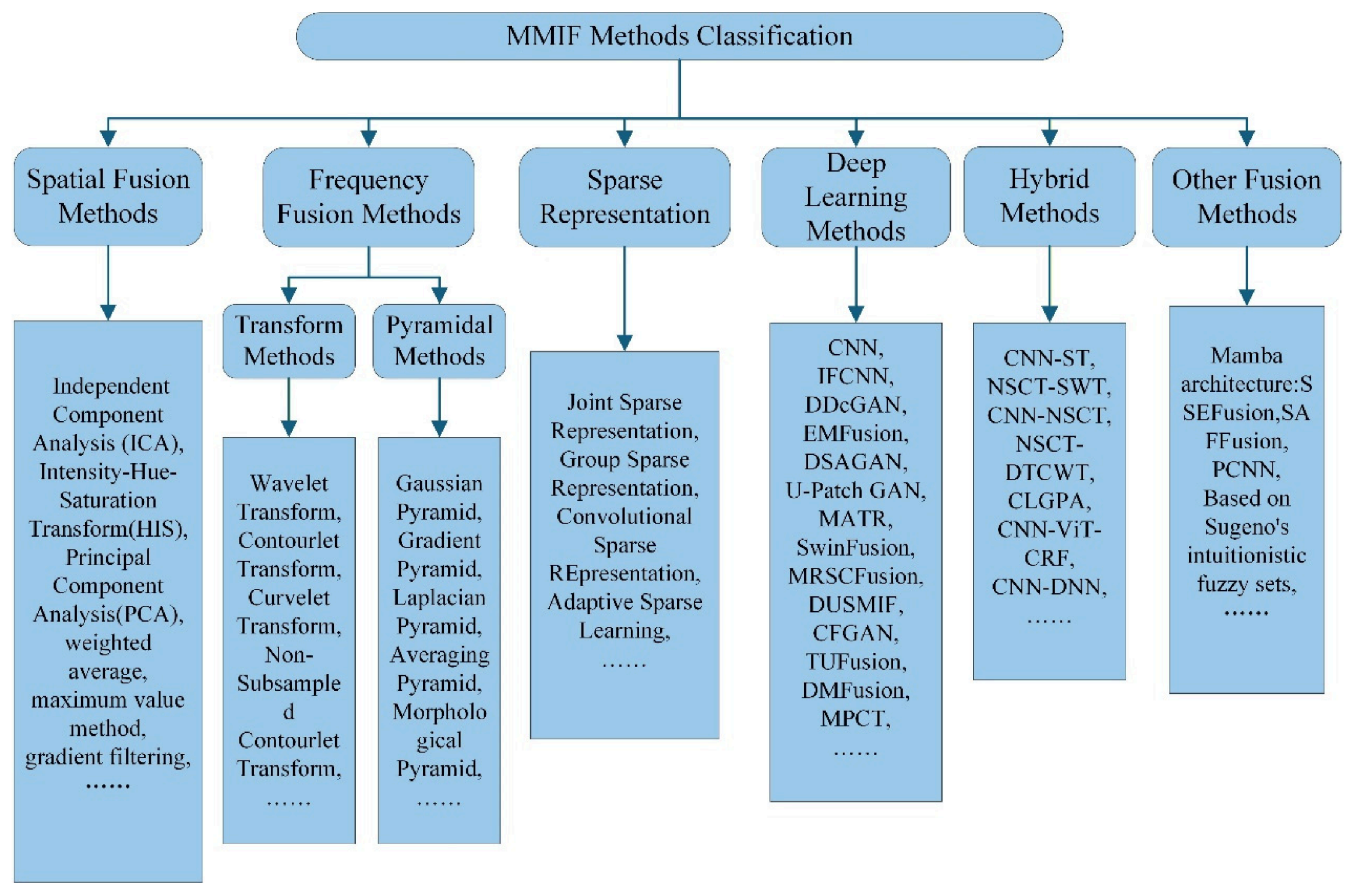
Classification of multimodal medical image fusion methods.

According to the fusion level, MMIF methods are further categorized into pixel-level, feature-level, and decision-level fusion, corresponding to increasing levels of information abstraction [12]. Pixel-level fusion directly combines source images, feature-level fusion integrates modality-specific features, and decision-level fusion merges the outputs of multiple decision-making systems. Representative methods include weighted averaging, ICA, PCA, wavelet transform, sparse representation, deep learning-based feature fusion, and fuzzy logic. The overall classification of MMIF methods is illustrated in Figure 6.

### 5.2. Spatial-Domain MMIF Methods

Spatial-domain methods perform pixel-level fusion by directly manipulating image pixel values rather than transform coefficients. Fusion is achieved by selecting pixels, blocks, or regions according to predefined saliency measures and combining them using linear or nonlinear strategies. Representative methods include minimum/maximum selection, arithmetic averaging, weighted averaging, Principal Component Analysis (PCA), Independent Component Analysis (ICA), and Intensity–Hue–Saturation (IHS) transformation [10].

Simple methods such as minimum/maximum selection, arithmetic averaging, and weighted averaging are computationally efficient but generally provide limited fusion performance. In contrast, ICA extracts statistically independent components from source images to separate complementary information from noise, whereas PCA projects correlated data onto orthogonal principal components to reduce redundancy while preserving the most informative features. IHS transformation performs fusion in the intensity component after converting images from the RGB color space into intensity, hue, and saturation components, followed by inverse transformation to reconstruct the fused image.

ICA- and PCA-based methods have been extensively studied in MMIF. For example, Vince D. Calhoun et al. [28] proposed an ICA-based feature fusion framework for functional MRI (fMRI) and MRI, while Panda et al. [29] incorporated the Escherichia coli Bacteria Foraging Optimization (ECBFO) algorithm to optimize ICA-derived components. Owing to its capability of reducing data redundancy and dimensionality, PCA has been widely adopted in image fusion applications [30]. Owing to its effectiveness in dimensionality reduction, PCA has been widely combined with other fusion techniques, including the Laplacian Pyramid [31], shearlet transform [32], two-dimensional PCA (2D-PCA) [33], Quaternion PCA (QPCA) [34], Stationary Wavelet Transform (SWT) [35], Singular Value Decomposition (SVD) [36], and Intensity–Hue–Saturation (IHS) transformation [37], resulting in improved multimodal medical image fusion performance.

Although spatial-domain methods are computationally efficient and easy to implement, they mainly exploit local pixel information and often fail to capture spatial contextual information and structural correlations, leading to spectral distortion and spatial deformation in the fused images. Consequently, recent research has increasingly focused on transform-domain and deep learning-based approaches, which are more effective in preserving structural details and complementary information. Nevertheless, spatial-domain methods remain attractive for applications requiring low computational complexity and real-time processing.

### 5.3. Transform-Domain MMIF Methods

To overcome the limitations of spatial-domain methods, transform-domain approaches first decompose source images into multiple sub-bands, fuse the corresponding transform coefficients according to predefined fusion rules, and then reconstruct the fused image through the inverse transform. Compared with spatial-domain methods, transform-domain techniques provide superior multiscale and directional representations, enabling better preservation of image structures and complementary information.

Transform-domain fusion methods can generally be divided into transform-based and pyramid-based techniques [24,38]. Transform-based methods mainly include Wavelet Transform (WT), Contourlet Transform (CT), Curvelet Transform (CVT), and their variants [25], whereas pyramid-based methods include the Gaussian Pyramid, Laplacian Pyramid (LP), Gradient Pyramid (GP), and Morphological Pyramid (MP). These methods provide multiscale image representations that effectively preserve spectral, structural, and texture information [39,40,41]. Among them, LP has been widely adopted because of its efficient multiscale decomposition capability. For example, Du et al. [40] employed LP for multiscale coefficient fusion, while Sahu et al. [42] further combined LP with the Discrete Cosine Transform (DCT) to enhance edge preservation and fusion quality.

Wavelet-based methods include the Discrete Wavelet Transform (DWT) [43], Redundant Discrete Wavelet Transform (RDWT) [44], Lifting Wavelet Transform (LWT) [45], and Multiwavelet Transform (MWT) [3,43]. These methods provide multiresolution representations of source images and have been extensively applied in MMIF [46]. Among them, DWT offers localization in both spatial and frequency domains but suffers from limited translation invariance and directional representation [47]. RDWT improves translation invariance by eliminating downsampling [48], MWT enhances detail and texture preservation through flexible filter-bank design, and LWT reduces computational complexity [49].

To further improve directional representation, several geometric transforms have been proposed. Contourlet-based methods, including the Contourlet Transform (CT), Dual-Tree Complex Contourlet Transform (DT-CCT), and Non-Subsampled Contourlet Transform (NSCT), provide efficient multiscale and multidirectional representations for preserving image contours and edges [44,50,51]. Curvelet-based methods comprise the Ridgelet Transform (RT) [50], Curvelet Transform (CVT) [51], Shearlet Transform (ST) [52], and Non-Subsampled Shearlet Transform (NSST) [45,53]. RT provides sparse representations of line singularities, CVT improves the representation of curved edges [54], ST enhances directional flexibility through shear operations [55], and NSST further suppresses pseudo-Gibbs artifacts while preserving translation invariance [50,51,52,53].

Numerous studies have combined transform-domain techniques with optimization algorithms to improve fusion performance. For example, Himanshi et al. [56] integrated CVT with Principal Component Analysis (PCA) for MRI–CT fusion, whereas Arif et al. [57] combined CVT with a Genetic Algorithm (GA) for multimodal fusion of MRI, MRA, PET, and SPECT images. Yin et al. [58] proposed an NSST-based framework in which a Parameter-Adaptive Pulse-Coupled Neural Network (PA-PCNN) was employed to fuse high-frequency coefficients, while Bhardwaj et al. [59] developed an NSCT-based multimodal MRI fusion framework using a GA-optimized Bayesian model.

Overall, transform-domain methods achieve superior multiscale and directional representations compared with spatial-domain approaches, enabling more effective preservation of image structures and complementary information. However, their performance is still influenced by transform selection and handcrafted fusion rules, limiting their ability to model complex nonlinear relationships across different imaging modalities.

### 5.4. Sparse Representation-Based MMIF Methods

Sparse Representation (SR) is an effective framework for modeling the characteristics of the human visual system. It represents source images using an overcomplete dictionary learned from image samples, enabling stable and discriminative image representations [60]. The fundamental idea of SR is to express an image signal as a linear combination of a small number of atoms selected from a pre-trained dictionary, where the corresponding sparse coefficients highlight the salient features of the input image. Sparsity refers to the use of only a few dictionary atoms during signal reconstruction, resulting in a sparse coefficient matrix [60]. Owing to its ability to efficiently represent image content while preserving essential structural information, SR has been widely applied in image processing and image fusion tasks.

Unlike traditional Multi-Scale Decomposition (MSD) methods, Sparse Representation (SR)-based fusion assumes that high- and low-frequency image components share the same sparse coefficients [61,62]. Originating from compressed sensing theory, SR has evolved into several variants, including Joint Sparse Representation (JSR) [63] and Group Sparse Representation (GSR) [64]. By exploiting the sparsity characteristics of image signals, these methods can effectively preserve important structural and textural information, thereby improving fusion performance. Liu et al. [65] proposed a fusion framework combining the Laplacian Pyramid (LP) and Convolutional Sparse Representation (CSR), aiming to overcome the limitations of local patch-based methods in preserving texture details.

### 5.5. Deep Learning-Based MMIF Methods

Over the past decade, deep learning (DL) has significantly advanced image classification [66], object detection, segmentation, and image fusion [67]. In recent years, numerous DL-based methods, particularly end-to-end frameworks, have been proposed for medical image fusion and have become a major research focus.

Compared with traditional fusion methods, DL-based models can automatically learn discriminative features from large-scale datasets and optimize feature representation through end-to-end training. Their flexible network architectures enable adaptive fusion of multimodal information, reducing the reliance on handcrafted fusion rules and manual parameter tuning. However, their performance remains highly dependent on the quality and quantity of the training data.

#### 5.5.1. Deep Learning-Based Image Fusion

The DL-based image fusion process generally consists of three stages: feature extraction, feature fusion, and image reconstruction [27]. In the feature extraction stage, deep neural networks are used to extract representative features or generate decision maps from source images, providing the basis for subsequent fusion. In the feature fusion stage, complementary features from multiple modalities are integrated using designed fusion strategies. Finally, the reconstruction stage maps the fused features back to the image domain to generate the final fused image, aiming to preserve complementary information and improve visual quality and diagnostic performance.

DL-based fusion methods can be broadly divided into end-to-end and non-end-to-end approaches [27]. End-to-end methods directly generate fused images from source images within a unified network, while non-end-to-end methods mainly use deep models for feature extraction or representation learning, followed by separate fusion and reconstruction steps.

Deep learning models learn hierarchical feature representations, where low-level features (e.g., edges and textures) are progressively transformed into high-level semantic representations (e.g., shapes and objects), enabling effective modeling of complex multimodal data [68,69].

Common DL-based fusion frameworks include Autoencoders (AEs), Convolutional Neural Networks (CNNs), Generative Adversarial Networks (GANs), and Transformers.

#### 5.5.2. CNN-Based Fusion Methods

CNN is one of the most widely used deep learning models in multimodal medical image fusion due to its strong capability in local feature extraction [70]. A typical CNN consists of stacked convolutional layers that generate feature maps through learnable kernels, combined with nonlinear activation and pooling operations to progressively learn hierarchical representations [71]. By capturing both low-level textures and high-level semantic features, CNNs are effective for modeling complex image structures in fusion tasks.

Early work by Liu et al. [72] first introduced CNNs into image fusion for multi-focus fusion, demonstrating improved detail and boundary preservation. Subsequently, CNN-based MMIF methods were developed using Siamese architectures to generate weight maps for feature integration [73,74], often combined with multi-scale pyramid decomposition and local similarity or comparison strategies to enhance fusion performance.

Zhang et al. [75] proposed IFCNN, a general CNN-based fusion framework that performs deep feature extraction and element-wise fusion, improving generalization and efficiency. Wang et al. [76] further combined Siamese CNN with contrast pyramid decomposition to enhance structural detail and contrast preservation.

To address information loss, Xu and Ma [77] proposed EMFusion, an unsupervised CNN-based framework with multi-level constraints for improved information preservation in CT–MRI, PET–MRI, and SPECT–MRI fusion. Lin et al. [78] introduced DUSMIF, a semantic-aware multi-branch CNN incorporating unsupervised segmentation and attention mechanisms to enhance feature extraction and fusion performance.

Although deep learning-based MMIF methods have achieved remarkable improvements in feature representation and fusion quality, their practical deployment is still constrained by computational complexity, memory consumption, and hardware requirements. CNN-based models generally provide efficient local feature extraction and relatively low computational costs, making them more suitable for real-time applications. However, their limited receptive fields may require deeper architectures or additional modules to capture global contextual information, increasing model complexity.

#### 5.5.3. GAN-Based Fusion Methods

Generative Adversarial Networks (GANs) model salient information in medical images through an adversarial learning mechanism. A typical GAN consists of two neural networks: a generator and a discriminator [7]. The generator learns the underlying distribution of source images to produce realistic synthetic images, while the discriminator aims to distinguish generated images from real ones. Through this adversarial training process, GANs can effectively enhance feature representation and preserve salient information, making them a promising framework for MMIF.

Ma et al. [79] proposed DDcGAN, which employs a dual-discriminator adversarial framework to preserve both functional and anatomical information in multimodal image fusion. Fu et al. [80] developed DSAGAN, a dual-stream attention-based GAN that enhances multimodal feature representation through multi-scale feature extraction and attention mechanisms.

#### 5.5.4. AE-Based Fusion Methods

Fan et al. [81] proposed U-Patch GAN, which employs a U-Net generator and a dual-adversarial PatchGAN framework to enhance high-frequency information preservation. The incorporation of dual adversarial learning and spectral normalization significantly improves fusion quality and detail retention.

Mao et al. [82] developed CFGAN for CT–MRI fusion. The framework adopts a dual-generator and dual-discriminator architecture and introduces discriminative feature extraction (DFE) and cross-dimensional interaction attention (CIA) modules to improve feature representation.

Fan et al. [83] proposed a semantic-aware medical image fusion framework, termed FW-Net. By integrating semantic constraints into an autoencoder-based architecture, the method alleviates semantic conflicts and improves fusion quality.

Ma et al. [84] introduced DMFusion, a dual-branch multi-scale feature fusion network for MMIF. The framework employs a dual-branch AE architecture to simultaneously learn modality-specific and modality-shared features.

#### 5.5.5. Transformer-Based Fusion Methods

Although CNNs provide an efficient and general framework for image fusion, their limited receptive fields restrict the modeling of long-range dependencies and global contextual information. In recent years, Transformer architectures, originally developed for natural language processing, have attracted increasing attention in MMIF due to their self-attention mechanism and superior capability for capturing global features and long-range dependencies. By projecting input features into query (Q), key (K), and value (V) representations, Transformers establish global relationships among features, thereby facilitating effective cross-modal information interaction and fusion [85].

Tang et al. [86] proposed an unsupervised Multi-scale Adaptive Transformer (MATR) for multimodal medical image fusion. The adaptive Transformer module enhances the extraction of global semantic information and cross-modal feature representation.

Ma et al. [87] introduced SwinFusion, a general image fusion framework based on Swin Transformer. By incorporating intra-domain self-attention and inter-domain cross-attention mechanisms, the framework effectively facilitates information interaction across different modalities. Zhao et al. [88] proposed Transformer-based Universal Fusion (TUFusion) to improve multi-domain image fusion. The method employs a hybrid Transformer–CNN autoencoder and a composite attention strategy to jointly exploit global and local information.

Transformer-based fusion methods introduce powerful global modeling capabilities through self-attention mechanisms, but the quadratic computational complexity and memory requirements associated with attention operations remain major limitations, particularly for high-resolution medical images. Although various efficient Transformer architectures have been proposed to reduce computational overhead, balancing global representation capability and resource efficiency remains challenging.

#### 5.5.6. Hybrid MMIF Methods

Due to the limitations of individual fusion methods in feature representation and information preservation, hybrid fusion approaches have attracted increasing attention. These methods integrate multiple techniques, such as spatial-domain, transform-domain, and deep learning-based approaches, to exploit complementary advantages and improve overall fusion performance.

Hermessi et al. [89] proposed a hybrid CNN–Shearlet Transform (ST) method based on Siamese feature learning for improved CT–MRI fusion and information preservation. Ramlal et al. [90] developed a framework combining NSCT and SWT to better preserve edge and detail information by leveraging their complementary properties. Wang et al. [91] introduced a CNN–NSCT model with PHF-CNN to enhance high-frequency feature preservation and fusion quality.

Alseelawi et al. [92] proposed a hybrid NSCT–DTCWT method, achieving improved image quality, efficiency, and visual fidelity compared with conventional transform-based approaches. Ramarao et al. [93] designed CLGPA, which integrates local Laplacian filtering, CNN mapping, and Gaussian reconstruction to balance fusion performance and computational cost. Mehta and Singh [94] developed a CNN–ViT hybrid framework for MRI–PET fusion, combining local feature extraction with long-range dependency modeling. Joy et al. [95] proposed multimodal fusion models that integrate early and late fusion strategies using CNNs, DNNs, and LSTMs for feature learning and classification.

### 5.6. Other Fusion Methods

#### 5.6.1. Fuzzy Logic-Based Methods

Zadeh first introduced Fuzzy Logic (FL) in 1965 [96], and Atanassov later extended it by proposing Intuitionistic Fuzzy Sets (IFS) [97]. Owing to the inherent uncertainty and blurred boundaries in medical images, fuzzy set theory (FST) has been widely applied to image fusion. FL employs membership functions and fuzzy rules to model uncertainty and can serve as both a feature representation and decision-making tool in MMIF [98,99]. Tirupal et al. [100] proposed a Sugeno-based intuitionistic fuzzy set approach for medical image fusion, which improves visualization quality and reduces uncertainty in the fused images.

#### 5.6.2. PCNN-Based Methods

In recent years, PCNNs have attracted considerable attention due to their ability to mimic information processing mechanisms in biological visual systems. Inspired by the visual cortex of mammals, PCNNs can effectively extract salient information from source images without a training process and have been widely applied in image fusion tasks [101,102]. Duan et al. [103] proposed a multimodal medical image fusion method combining PCNN and low-rank representation. The method employs NSCT for multi-scale decomposition, applies a K-SVD-based low-rank fusion strategy to low-frequency sub-bands, and utilizes PCNN for high-frequency fusion, thereby improving detail preservation in the fused images.

#### 5.6.3. Mamba-Based Methods

CNNs are effective at extracting local features, whereas Transformers excel at modeling global dependencies but suffer from high computational complexity. To address this issue, State Space Models (SSMs) have been introduced as efficient architectures for long-range context modeling [104]. Building upon SSMs, Mamba [105] incorporates a selective state space mechanism to enhance long-range dependency modeling, while VMamba [106] extends this framework to vision tasks and demonstrates strong global feature modeling capability [107,108]. Recently, Mamba has been introduced into MMIF. Liu et al. [109] proposed SAFFusion, which combines Mamba with contourlet transform for efficient multi-scale frequency-domain feature extraction. Subsequently, Liu et al. [110] developed SSEFusion, integrating Mamba and spiking neural networks (SNNs) to enhance salient semantic feature extraction and improve fusion performance.

Recently, Mamba-based architectures have attracted increasing attention due to their linear computational complexity and efficient long-range dependency modeling. Nevertheless, their practical advantages in MMIF still require comprehensive validation, including comparisons of inference speed, memory consumption, energy efficiency, and deployment feasibility on clinical hardware platforms.

Therefore, future MMIF research should consider not only fusion accuracy but also computational efficiency, energy consumption, and hardware adaptability to facilitate real-world clinical implementation.

Finally, Table 3 summarizes the representative studies and key characteristics of the aforementioned MMIF methods.

### 5.7. Comparative Analysis, Practical Considerations, and Limitations of Existing MMIF Methods

Although existing MMIF methods have achieved significant progress, their practical applicability varies depending on computational resources, data availability, interpretability, and clinical requirements. Conventional methods, including spatial-domain, transform-domain, and sparse representation-based approaches, generally provide efficient implementation and good interpretability. However, their performance relies on handcrafted rules, transformation strategies, or predefined dictionaries, limiting their adaptability to complex medical images.

Deep learning-based methods improve feature representation and fusion performance through automatic feature learning. Nevertheless, their effectiveness depends strongly on training data quality and diversity, and they may suffer from overfitting, dataset bias, and limited generalization across different imaging conditions. Transformer- and Mamba-based methods further enhance global feature modeling but introduce challenges related to computational cost, memory consumption, interpretability, and clinical deployment.

Therefore, fusion quality alone is insufficient for evaluating MMIF methods. Practical factors, including robustness, efficiency, generalization capability, and clinical feasibility, should also be considered. No single strategy is optimal for all scenarios: conventional methods remain suitable for lightweight and interpretable applications, whereas deep learning-based methods are preferred when sufficient data and computational resources are available. Table 4 summarizes the advantages, limitations, and practical considerations of representative MMIF methods.

## 6. Image Fusion Quality Assessment

Quality assessment plays a crucial role in evaluating the effectiveness of image fusion algorithms. Existing evaluation methods can generally be categorized into subjective and objective assessment. Subjective assessment compares fused images with source images through visual inspection, focusing on detail preservation, structural integrity, and overall visual quality. However, it is highly dependent on human judgment and is often time-consuming and labor-intensive [11]. In contrast, objective assessment quantitatively evaluates fusion performance using mathematical metrics, providing better repeatability and reliability. Depending on the availability of reference images, objective metrics can be further divided into full-reference and no-reference categories. Since ground-truth fused images are generally unavailable in MMIF, no-reference metrics have become the most widely adopted evaluation tools in this field [13].

### 6.1. Subjective Evaluation

Subjective evaluation assesses fusion quality based on human visual perception and is widely adopted due to its simplicity and consistency with clinical diagnostic practices. A common approach is to collect scores from multiple observers through questionnaires and perform statistical analysis on the results [123]. However, the evaluation outcomes may be influenced by factors such as observer experience, mental condition, and visual acuity, which can reduce consistency and objectivity [124]. Therefore, subjective assessment is often combined with objective metrics to provide a more comprehensive and reliable evaluation of fusion performance.

### 6.2. Objective Evaluation

Objective evaluation quantitatively assesses fusion performance using mathematical models, providing reliable and reproducible results. To better reflect human visual perception of image quality, numerous evaluation metrics have been developed and selected according to different fusion tasks and application scenarios. Based on the availability of reference images, objective metrics can generally be categorized into full-reference and no-reference metrics [3].

#### 6.2.1. Full-Reference Metrics

Full-reference evaluation metrics assess fusion quality by comparing the fused image with source images or a reference image (ground truth). These metrics are primarily used to measure the amount of information transferred from the source images to the fused image and to evaluate the correlation between them. Depending on the application scenario, the reference image may be either the source image itself or a predefined ground-truth image. This section summarizes representative full-reference metrics, including Mutual Information (MI), Root Mean Square Error (RMSE), Peak Signal-to-Noise Ratio (PSNR), Structural Similarity Index Measure (SSIM), Correlation Coefficient (CC), Nonlinear Correlation Information Entropy (NCIE), Gradient-Intensity Mixed Information (GIMI), and Visual Information Fidelity (VIF).

(1) Mutual Information (MI)

Mutual information measures the amount of information transferred from the reference image to the fused image and reflects the shared information between them [125]. A larger MI value indicates that more information, particularly detail and texture information, is preserved from the source image in the fused image, thereby implying better fusion performance [11]. It is commonly used to evaluate how well the fusion process retains information from the source images. It is defined as:

(1) $$MI=MI_{A,F}+MI_{B,F}$$

where $MI_{A,F}$ and $MI_{B,F}$ denote the mutual information between source images A and B and the fused image F, respectively, reflecting the amount of information transferred from the source images to the fused image. The expression is given as:

(2) $$MI_{X,F}=\sum_{x,f}p_{X,F}(x,f)\log_{2}\frac{p_{X,F}(x,f)}{p_{X}(x)p_{F}(f)}$$

where $p_{X}(x)$ and $p_{F}(f)$ denote the marginal histograms of the source image X and the fused image F, respectively, and $p_{X,F}(x,f)$ represents their joint histogram.

(2) Root Mean Square Error (RMSE)

Root Mean Square Error measures the pixel-wise difference between the fused image and the reference image, thereby quantifying the deviation between them and evaluating the reconstruction accuracy of the fusion result [124]. A lower RMSE value indicates that the fused image is closer to the reference image, implying better fusion performance. It is defined as:

(3) $$RMSE=\frac{RMSE_{A,F}+RMSE_{B,F}}{2}$$

(4) $$RMSE=\sqrt{\frac{1}{MN}\sum_{i=0}^{M-1}\sum_{j=0}^{N-1}(X(i,j)-F(i,j){)}^{2}}$$

(3) Peak Signal-to-Noise Ratio (PSNR)

Peak Signal-to-Noise Ratio is a widely used metric for evaluating the overall quality of image fusion results [126]. It measures the reconstruction fidelity of the fused image with respect to the reference image by utilizing the mean squared error (MSE) between corresponding pixel intensities. A higher PSNR value indicates lower distortion and better fusion quality. It is defined as:

(5) $$PSNR=10\mathrm{lg}\frac{r^{2}}{MSE}$$

where r denotes the maximum pixel intensity of the fused image, MSE and represents the Mean Squared Error, which is the square of the Root Mean Square Error (RMSE). It measures the average squared difference between corresponding pixels of the reference image and the fused image. The MSE is calculated as:

(6) $$MSE=\frac{MSE_{A,F}+MSE_{B,F}}{2}$$

(7) $$MSE_{X,F}=\frac{1}{MN}\sum_{i=0}^{M-1}\sum_{j=0}^{N-1}(X(i,j)-F(i,j){)}^{2}$$

(4) Structural Similarity Index Measure (SSIM)

SSIM is used to evaluate the structural similarity between the reference image and the fused image [127]. By modeling human visual perception, it assesses image quality from three aspects: luminance, contrast, and structural information. Compared with error-based metrics, SSIM can more effectively reflect information preservation and distortion introduced during the fusion process. A higher SSIM value indicates greater similarity between the fused image and the reference image and, consequently, better fusion performance. The SSIM between the source image X and the fused image F is defined as:

(8) $$SSIM_{X,F}=\sum_{x,f}\frac{2\mu_{x}\mu_{f}+C_{1}}{\mu_{x}^{2}+\mu_{f}^{2}+C_{1}}\times \frac{2\sigma_{x}\sigma_{f}+C_{2}}{\sigma_{x}^{2}+\sigma_{f}^{2}+C_{2}}\times \frac{2\sigma_{x,f}+C_{3}}{\sigma_{x}\sigma_{f}+C_{3}}$$

where $\mu_{x}$ and $\mu_{f}$ denote the mean intensities of the source image X and the fused image F, respectively; $\sigma_{x}$ and $\sigma_{f}$ represent their standard deviations; and $\sigma_{x,f}$ denotes the covariance between them. The constants $C_{1}$, $C_{2}$, and $C_{3}$ are introduced to stabilize the division operation and avoid numerical instability when the denominator approaches zero.

(5) Correlation Coefficient (CC)

The correlation coefficient measures the similarity between the reference image and the fused image, reflecting their structural consistency and shared information [128]. A higher CC value indicates that more information from the source image is preserved in the fused image, implying better fusion performance. It is defined as:

(9) $$CC=\frac{2C_{rf}}{C_{r}+C_{f}}$$

where $C_{rf}$ denotes the cross-correlation between the reference image and the fused image, while $C_{r}$ and $C_{f}$ represent the auto-correlation of the reference image and the fused image, respectively.

(6) Nonlinear Correlation Information Entropy (NCIE)

Nonlinear Correlation Information Entropy (NCIE) is used to measure the nonlinear correlation among source images A, B, and the fused image F [129]. It constructs a nonlinear correlation matrix R based on the nonlinear correlation coefficients between the source images and the fused image, which reflects the dependency relationships among multiple images. It is defined as:

(10) $$R=\left[\begin{array}{ccc} 1 & NCC_{A,B} & NCC_{A,F}\\ NCC_{B,A} & 1 & NCC_{B,F}\\ NCC_{F,A} & NCC_{F,A} & 1 \end{array}\right]$$

The NCIE is defined as:

(11) $$NCIE=1+\sum_{i=1}^{3}\frac{\lambda_{i}}{3}\log_{256}\frac{\lambda_{i}}{3}$$

where $\lambda_{i}$ denotes the i-th eigenvalue of the nonlinear correlation matrix R. A larger NCIE value indicates a stronger nonlinear correlation between the source images and the fused image, implying that more useful information from the source images is preserved in the fused image and, consequently, better fusion performance.

(7) Gradient-Intensity Mixed Information (GIMI)

GIMI is an image quality assessment metric derived from mutual information. It combines gradient and intensity information to evaluate the spatial similarity between the source image and the fused image [130]. By jointly considering intensity distributions, edge characteristics, and structural information, GIMI provides a more comprehensive assessment of information preservation in the fused image. A higher GIMI value indicates that the fused image preserves more intensity and structural information from the source images, resulting in better fusion performance.

It is defined as:

(12) $$GIMI=2\left(\frac{H_{ig}(A,F)}{H_{ig}(A)+H_{ig}(F)}+\frac{H_{ig}(B,F)}{H_{ig}(B)+H_{ig}(F)}\right)$$

where $H_{ig}(A,F)$ and $H_{ig}(B,F)$ denote the joint entropy between source images A, B and the fused image F, respectively, while $H_{ig}(A)$, $H_{ig}(B)$, and $H_{ig}(F)$ represent the entropy of the corresponding images.

(8) Visual Information Fidelity (VIF)

VIF is an image quality assessment metric based on natural scene statistics and the human visual system. It measures the amount of visual information preserved in the fused image with respect to the reference image [131]. By quantifying the shared visual information between the reference image and the fused image, VIF effectively evaluates the preservation of structural and detail information during the fusion process. A higher VIF value indicates better visual information preservation and superior fusion performance. It is defined as:

(13) $$VIF=\sum_{i=1}^{N}p_{i}\cdot \mathrm{VIF}(A,B,F)=\;\sum_{i=1}^{N}p_{i}\frac{\sum_{b}FVID_{i,b}(A,B,F)}{\sum_{b}FVIND_{i,b}(A,B,F)}$$

where FVID and FVIND denote the distorted visual information and the reference visual information, respectively, and $p_{i}$ represents the weighting coefficient of the corresponding sub-band.

These metrics provide objective measurements by evaluating similarity or information consistency with source/reference images, but they may not fully reflect the perceptual or clinical significance of fused results.

#### 6.2.2. No-Reference Metrics

No-reference evaluation metrics assess fusion quality without requiring a reference image and rely solely on information contained in the fused image. These metrics are mainly used to measure the information content, detail richness, and sharpness of the fused image and are therefore widely adopted in medical image fusion tasks where ground-truth images are unavailable. This section summarizes commonly used no-reference metrics, including Entropy (EN), Average Gradient (AG), Standard Deviation (SD), Spatial Frequency (SF), Chen–Blum Metric (CB), Cross Entropy (CE), and Edge Intensity (EI).

(9) Entropy (EN)

Entropy is one of the most widely used no-reference image quality assessment metrics and is employed to measure the amount of information contained in the fused image [132]. A higher EN value indicates that the fused image contains more information and preserves more useful details from the source images. It is defined as:

(14) $$EN=\sum_{l=0}^{L-1}p_{l}\log_{2}p_{l}$$

where L denotes the number of gray levels, and $p_{l}$ represents the normalized histogram probability of the gray level in the fused image.

(10) Average Gradient (AG)

AG is used to measure the average rate of gray-level variation in the fused image and reflects its sharpness, texture richness, and detail preservation capability [124]. Images containing more edges and texture information generally exhibit higher AG values. It is defined as:

(15) $$AG=\frac{1}{MN}\sum_{i=1}^{M}\sum_{j=1}^{N}\sqrt{\frac{\nabla F_{x}^{2}(i,j)+\nabla F_{y}^{2}(i,j)}{2}}$$

where $\nabla F_{x}(i,j)=F(i,j)-F(i+1,j)$, $\nabla F_{y}(i,j)=F(i,j)-F(i,j+1)$.

(11) Standard Deviation (SD)

SD measures the dispersion of gray-level intensity values in the fused image, reflecting its contrast and distribution characteristics [133]. A higher SD value indicates greater intensity variation, higher contrast, and richer visual information in the fused image, which generally corresponds to better fusion performance. It is defined as:

(16) $$SD=\sqrt{\sum_{i=1}^{M}\sum_{j=1}^{N}(F(i,j)-\mu {)}^{2}}$$

where μ denotes the mean gray value of the fused image.

(12) Spatial Frequency (SF)

SF measures the rate of gray-level variations in the fused image and reflects its overall sharpness as well as the richness of texture and detail information [134]. It evaluates image quality by quantifying the intensity variations in the horizontal and vertical directions. Generally, a higher SF value indicates that the fused image contains more texture details and structural information, resulting in better fusion performance. It is defined as:

(17) $$SF=\sqrt{RF^{2}+CF^{2}}\;$$

where RF and CF denote the row frequency and column frequency, respectively. The corresponding formulations are given as follows:

(18) $$RF=\sqrt{\sum_{i=1}^{M}\sum_{j=1}^{N}(F(i,j)-F(i,j-1){)}^{2}}$$

(19) $$CF=\sqrt{\sum_{i=1}^{M}\sum_{j=1}^{N}(F(i,j)-F(i-1,j){)}^{2}}$$

(13) Chen–Blum Metric (CB)

CB evaluates the quality of the fused image by simulating the perception process of the Human Visual System (HVS) for image contrast and salient features [135]. This metric comprehensively considers local contrast, salient regions, and visual perceptual characteristics, thereby providing an effective assessment of the perceptual quality of the fused image. In general, a higher CB value indicates greater consistency with human visual perception and better fusion performance. It is defined as:

(20) $$CB=\frac{1}{MN}\left(\sum_{i=1}^{N}\sum_{j=1}^{M}\beta_{A}(i,j)W_{A,F}(i,j)+,\beta_{B}(i,j)W_{B,F}(i,j)\right)\;$$

where $W_{A,F}(i,j)$ and $W_{B,F}(i,j)$ denote the contrast information of the source image and the fused image, respectively, while $\beta_{A}$ and $\beta_{B}$ represent their corresponding saliency maps.

(14) Cross Entropy (CE)

CE is an information-theoretic metric used to quantify the difference in intensity distributions between the reference image and the fused image [136,137]. In general, a smaller CE value indicates a lower information difference between the reference image and the fused image, implying better fusion performance. It is defined as:

(21) $$CE=\frac{CE_{A.F}+CE_{B.F}}{2}$$

where X denotes the source image A or B, and $CE_{X,F}$ represents the cross entropy between the source image X and the fused image F. The overall cross entropy can be expressed as:

(22) $$CE_{X,F}=\sum_{i=0}^{255}h_{X}(i)\log_{2}\frac{h_{X}(i)}{h_{F}(i)}$$

where $h_{X}(i)$ denotes the normalized histogram probability distribution of the corresponding image.

(15) Edge Intensity (EI)

EI is a gradient-based no-reference image quality metric used to quantify the strength of edge information in the fused image [138]. Since edge features represent important structural and detail information, EI is commonly computed using the Sobel operator to extract image gradients and evaluate edge preservation capability. A higher EI value indicates clearer edge structures and richer detail information in the fused image, implying better fusion performance. It is defined as:

(23) $$EI=\sqrt{S_{x}^{2}+S_{y}^{2}}$$

where $S_{x}=F^{*}h_{x},S_{y}=F^{*}h_{y},h_{x}=\left[\begin{array}{ccc} -1 & 0 & 1\\ -2 & 0 & 2\\ -1 & 0 & 1 \end{array}\right],h_{y}=\left[\begin{array}{ccc} -1 & -2 & -1\\ 0 & 0 & 0\\ 1 & 2 & 1 \end{array}\right]$.

Although no-reference metrics avoid the requirement for reference images, they mainly evaluate intrinsic image characteristics and may be sensitive to noise, artifacts, or artificial enhancement.

Table 5 presents a quantitative comparison of the representative deep learning-based multimodal medical image fusion methods introduced in the previous section using multiple evaluation metrics.

### 6.3. Limitations of Current Evaluation Metrics and Clinical Validation

Although existing evaluation metrics provide quantitative measurements of fused image quality, their correlation with clinical diagnostic performance remains insufficiently explored. Most commonly used metrics, such as MI, PSNR, SSIM, VIF, and EN, quantify different aspects of fusion quality, including information preservation, structural consistency, visual fidelity, and image contrast. For example, information-based metrics evaluate the amount of complementary information retained from source images, whereas structural and perceptual metrics focus more on spatial consistency and human visual characteristics. However, higher metric values do not necessarily indicate that fused images provide more clinically meaningful information or improve diagnostic decision-making.

A major limitation of current evaluation strategies is the lack of clinical validation. For example, metrics that favor enhanced contrast or sharpness may not always correspond to better lesion visualization or improved interpretation by clinicians. Therefore, objective image quality assessment should be complemented by clinically oriented evaluations, including expert assessment, multi-reader studies, and downstream tasks such as lesion detection, segmentation, and classification.

Future MMIF studies should establish more comprehensive evaluation frameworks that integrate conventional image quality metrics with clinical validation. Multi-center datasets and task-oriented evaluations are also needed to better assess the generalizability and practical value of fusion methods in real-world clinical scenarios.

## 7. Challenges and Future Research Directions

### 7.1. Current Challenges

Multimodal medical image fusion (MMIF) has achieved significant progress in integrating complementary information from different imaging modalities. However, its clinical translation remains limited by challenges related to data availability, image registration, fusion strategies, evaluation standards, and computational efficiency.

The limited availability of large-scale, diverse, and clinically representative medical imaging datasets remains a major challenge. Existing studies are often evaluated on small public datasets collected under specific acquisition protocols, which may introduce dataset bias and limit model generalization across different scanners, institutions, and clinical scenarios. Moreover, the lack of reliable ground-truth fused images makes it difficult to establish evaluation criteria that accurately reflect clinical value.

Image registration is another critical issue affecting MMIF performance. Differences in acquisition protocols, patient motion, and anatomical deformation may cause spatial misalignment between multimodal images, and registration errors can propagate into subsequent fusion processes. In addition, clinical images may contain noise, intensity variations, and missing modalities, requiring robust fusion frameworks capable of handling noisy, misaligned, and incomplete multimodal data under realistic clinical conditions.

The selection of appropriate fusion strategies remains challenging because different approaches exhibit distinct trade-offs among representation capability, computational efficiency, interpretability, and clinical applicability. Conventional methods rely on handcrafted features, transformation strategies, or predefined fusion rules, whereas deep learning-based methods provide powerful feature representation capabilities but may suffer from overfitting, limited interpretability, and poor cross-domain generalization. Meanwhile, advanced architectures such as Transformer- and Mamba-based models introduce additional concerns regarding computational cost, memory requirements, and clinical deployment.

Furthermore, the lack of standardized evaluation protocols restricts fair comparisons among MMIF methods. Different datasets, preprocessing procedures, registration strategies, and evaluation metrics make it difficult to assess the true advantages of competing approaches. In particular, commonly used image quality metrics mainly measure information preservation and structural similarity, while their correlation with clinical diagnosis remains insufficiently validated.

### 7.2. Future Research Directions

Future MMIF research should move beyond improving image quality metrics and focus on developing robust, efficient, and clinically oriented fusion frameworks. Task-aware fusion algorithms represent an important direction, where fusion strategies are designed according to specific clinical objectives, such as diagnosis, lesion detection, segmentation, and treatment planning. Incorporating knowledge of imaging mechanisms and acquisition characteristics into fusion models may further improve the preservation of modality-specific and clinically meaningful information.

The development of standardized datasets and evaluation frameworks is also essential for advancing MMIF toward clinical applications. Future studies should establish large-scale, multi-center datasets covering diverse anatomical structures, pathological conditions, and acquisition settings. Meanwhile, evaluation protocols should combine objective image quality metrics with clinical assessments and downstream medical tasks to provide a more comprehensive measurement of fusion effectiveness and clinical relevance.

Improving computational efficiency and deployment feasibility will remain an important research direction. Although advanced architectures, including Transformer- and Mamba-based models, provide powerful feature representation capabilities, their computational costs and memory requirements may limit practical applications. Therefore, lightweight and efficient fusion networks should be explored to enable real-time deployment in resource-constrained clinical environments.

Recent advances in foundation models, vision-language models (VLMs), and large multimodal models (LMMs) provide new opportunities for MMIF by enabling cross-modal representation learning from large-scale heterogeneous biomedical data. Compared with conventional task-specific fusion networks, these models have the potential to improve adaptability and generalization across different imaging scenarios. However, their application in MMIF remains at an early stage and faces challenges including high computational requirements, limited interpretability, data privacy concerns, and insufficient clinical validation. Future studies should investigate how these emerging models can be effectively integrated into MMIF frameworks while maintaining computational efficiency, interpretability, data security, and clinical reliability.

Ultimately, the successful translation of MMIF into clinical practice will require coordinated progress in algorithm design, data resources, evaluation standards, and clinical validation. By addressing these challenges, future fusion methods are expected to achieve improved robustness, generalization capability, and practical utility in real-world medical scenarios.

## 8. Conclusions

This study provides a comprehensive review of multimodal medical image fusion (MMIF) by systematically summarizing existing research from both technical and clinical perspectives. Various imaging modalities commonly used in MMIF, as well as emerging modalities with potential applications, are introduced and analyzed according to their data characteristics, acquisition mechanisms, and complementary information.

In addition, publicly available multimodal medical imaging databases are reviewed, and their characteristics, applications, and limitations are discussed. The general workflow of MMIF and representative fusion strategies are summarized, including spatial-domain methods, transform-domain methods, sparse representation-based methods, conventional approaches, deep learning-based methods, hybrid strategies, and emerging Mamba-based approaches. Particular emphasis is placed on recent advances in deep learning-based fusion methods and their potential advantages and challenges.

Furthermore, commonly used image fusion evaluation metrics are reviewed, and the reported performance of representative MMIF methods is summarized and analyzed. Beyond algorithmic development, this review discusses critical challenges, including dataset limitations, robustness under clinical uncertainties, interpretability, computational efficiency, and clinical translation. From a clinical perspective, the practical value of MMIF is not determined solely by quantitative image quality metrics, but also by its reliability, generalizability, and applicability in real-world scenarios.

As a review article, this work synthesizes existing evidence rather than introducing new experimental results. Future research should focus on developing more robust, efficient, and clinically applicable MMIF frameworks, while establishing standardized clinical validation protocols. In addition, emerging multimodal learning paradigms, including the integration of medical images with physiological signals, sensor data, and large-scale multimodal models, may provide new opportunities for advancing future medical fusion systems.

## Figures and Tables

**Table 1:** Classification and characteristics of major imaging modalities involved in multimodal medical image fusion (MMIF).

MMIF Imaging Modality	Full Name	Invasive/ Non-Invasive	Characteristics
X-Ray	X-ray Imaging	Non-invasive	Provides fast and low-cost anatomical imaging, particularly suitable for visualizing bone structures, fractures, and calcifications.
MRI	Magnetic Resonance Imaging	Non-invasive	Offers excellent soft-tissue contrast and high spatial resolution, enabling detailed visualization of anatomical structures and pathological tissues.
CT	Computed Tomography	Non-invasive	Provides high-resolution cross-sectional anatomical images and is particularly effective for imaging bones, organs, and acute hemorrhages.
PET	Positron Emission Tomography	Non-invasive (radiotracer-assisted)	Provides functional and metabolic information by visualizing radiotracer uptake, widely used in oncology, neurology, and cardiology.
SPECT	Single-Photon Emission Computed Tomography	Non-invasive (radiotracer-assisted)	Evaluates regional blood flow and physiological functions using gamma-emitting radiotracers, supporting neurological and cardiovascular diagnosis.
US	Ultrasound	Non-invasive	Produces real-time images of soft tissues and blood flow without ionizing radiation, making it suitable for dynamic examinations.
Microscopy	Microscopy	Typically Ex Vivo/In Vitro	Provides micrometer- to nanometer-scale structural information for cellular, tissue, and subcellular analysis.
OCT	Optical Coherence Tomography	Non-invasive	Generates high-resolution cross-sectional images of biological tissues, particularly useful in ophthalmology and dermatology.
Endoscopy	Endoscopy	Invasive/Minimally Invasive	Uses an endoscope inserted through natural body openings or small incisions to visualize internal organs and tissue surfaces directly.
MRA	Magnetic Resonance Angiography	Non-invasive	A specialized MRI technique for vascular imaging, enabling visualization of blood vessels and blood flow without ionizing radiation.
DSA	Digital Subtraction Angiography	Invasive	Provides high-resolution vascular imaging through catheter-based contrast agent injection and is considered the clinical standard for vascular assessment.

**Table 2:** Comparison of publicly available multimodal medical imaging databases used in MMIF research.

Database	Release Year	Imaging Modalities	Anatomical Region	Characteristics	Data Format
OASIS	2007 (OASIS-1), 2009 (OASIS-2), 2019 (OASIS-3)	MRI, PET (OASIS-3)	Brain	OASIS-1 contains cross-sectional MRI data from 416 subjects. OASIS-2 provides longitudinal MRI data from 150 subjects. OASIS-3 includes MRI and PET data from more than 1000 participants, supporting studies of aging and Alzheimer’s disease.	DICOM, NIfTI
AANLIB	1995	MRI, CT, PET, SPECT	Brain	Contains normal and pathological brain images, including stroke, tumors, degenerative disorders, and infectious diseases. Multimodal images are generally provided in a spatially aligned form.	GIF
TCIA	2011	CT, MRI, PET, PET/CT, Mammography, X-ray, US, SPECT, etc.	Brain, lung, breast, colon, kidney, chest, abdomen, head and neck, etc.	One of the largest public cancer imaging repositories, providing multimodal imaging data together with clinical outcomes, genomic information, pathology reports, and expert annotations.	DICOM
ADNI	2004	MRI, PET, fMRI	Brain	Focuses on normal aging, mild cognitive impairment (MCI), and Alzheimer’s disease. Provides longitudinal neuroimaging, clinical, genetic, and biomarker data.	DICOM, NIfTI, CSV
MIDAS(RIRE)	1998–2000 (RIRE Project)	CT, MRI, PET	Brain, head	Primarily designed for multimodal image registration and analysis. Includes pre-aligned CT, MRI, and PET images widely used in image registration and fusion studies.	DICOM
OCTA-500	2021	OCT, OCTA	Retina	Contains OCT and OCTA data from 500 subjects. Includes two FOV settings (3 mm × 3 mm and 6 mm × 6 mm), six projection maps, textual annotations, and multiple segmentation labels.	PNG, MAT

**Table 3:** Representative studies on MMIF and their technical categories.

Research Work	Fusion Technology Field	Year	Image Modality	MMIF Technology	Body Organ	Dataset
Calhoun et al. [28]	Spatial-Domain MMIF Methods	2009	f-MRI/MRI	ICA	brain	—
He et al. [37]		2010	MRI/PET	HIS-PCA	brain	AANLIB
Bashir et al. [35]		2019	CT/MRI/X-rays	SWT-PCA	brain, leg	—
Faragallah et al. [36]		2021	CT/MRI	PCA-SVD	brain	AANLIB
Rehal et al. [111]		2021	MRI/PET	2-DHT-HIS	brain	—
Bhatnagar et al. [112]	Transform-Domain MMIF Methods	2013	CT/MRI-T1/MRI-T2	NSCT	brain	AANLIB
Himanshi et al. [56]		2016	CT/MRI	PCA-CVT	brain	AANLIB
Zhu et al. [113]		2019	CT/MRI MRI/PET SPECT/MRI	NSCT-local Laplacian energy	brain	AANLIB
Yin et al. [58]		2019	MRI/CT/PET	parameter-adaptive PCNN-NSST	brain	ADNI
Arif et al. [57]		2019	MRA/MRI PET/SPECT	CVT through genetic algorithm	brain	AANLIB
Polinat et al. [114]		2020	MRI/PET/SPECT	Empirical Wavelet Decomposition	brain	AANLIB
Hu et al. [115]		2020	CT/MRI MR-T1/MR-T2	Separable dictionary learning and Gabor filtering-NSCT	brain	AANLIB
Bhardwaj et al. [59]		2020	MR-T1/MR-T2	NSCT in Genetic algorithm optimization	brain	AANLIB
Wang et al. [116]		2022	SPECT-T1/SPECT-TC	DCT in geometric algebra	brain	AANLIB
Qiu et al. [117]	Sparse Representation-Based MMIF Methods	2017	CT/MRI	NSCT-SR	brain	AANLIB
Li et al. [118]		2018	CT/MRI	NSCT and SR	brain	AANLIB
Liu et al. [65]		2020	CT/MRI	Laplacian pyramid and convolutional SR	brain	Imagefusion.org
Maqsood et al. [119]		2020	CT/MRI	Two-scale Image Decomposition-SR	brain	—
Polinat et al. [120]		2021	CT/MRI	adaptive SR	brain	AANLIB
Rajalingam et al. [74]	Deep Learning-Based MMIF Methods	2018	CT/MRI MRI/PET	CNN	brain	AANLIB Radiopedia.org
Zhang et al. [75]		2020	CT/MRI	IFCNN	brain	—
Ma et al. [79]		2020	MRI/PET	DDcGAN	brain	—
Xu et al. [77]		2021	CT/MRI PET/MRI SPECT/MRI	EMFusion	brain	AANLIB
Fu et al. [80]		2021	MRI/PET MRI/SPECT	DSAGAN	brain	AANLIB
Fan et al. [81]		2022	CT/MRI MRI/SPECT PET/MRI	U-Patch GAN	brain	AANLIB
Tang et al. [86]		2022	MRI/SPECT MRI/PET	MATR	brain	AANLIB
Ma et al. [87]		2022	CT/MRI MRI/PET	SwinFusion	brain	AANLIB
Xie et al. [121]		2023	MRI/CT MRI/PET MRI/SPECT	MRSCFusion	brain	AANLIB
Lin et al. [78]		2024	CT/MRI PET/MRI	DUSMIF	brain	AANLIB
Mao et al. [82]		2024	CT/MRI	CFGAN	brain	AANLIB
Zhao et al. [88]		2024	CT/MRI MRI/PET	TUFusion	brain	AANLIB
Ma et al. [84]		2025	MRI/CT MRI/PET MRI/SPECT	DMFusion	brain	AANLIB
Xu et al. [122]		2025	MRI/CT MRI/PET MRI/SPECT	MPCT	brain	AANLIB
Ramarao et al. [93]	Hybrid MMIF Methods	2024	MRI/CT	CLGPA	brain	AANLIB
Mehta et al. [94]		2024	MRI/PET	CNN-ViT-CRF	brain	AANLIB
Joy et al. [95]		2025	MRI/DaT	CNN-DNN	brain	NTUA
Tirupal et al. [100]	Other Fusion Methods	2017	MRI/CT MRI/MRA MRI/PET MRI/SPECT X-rays/VA	Based on Sugeno’s intuitionistic fuzzy sets	brain	AANLIB
Duan et al. [103]		2022	MRI/PET	PCNN	brain	AANLIB
Liu et al. [110]		2025	MRI/CT MRI/PET MRI/SPECT	SSEFusion	brain	AANLIB
Liu et al. [109]		2025	MRI/CT MRI/PET MRI/SPECT	SAFFusion	brain	AANLIB

**Table 4:** Comparative analysis of representative MMIF methods.

Method Category	Main Advantages	Major Limitations	Generalization and Clinical Concerns	Suitable Application Scenarios
Spatial-domain methods	Simple implementation; low computational cost; effective spatial detail preservation	Depend on handcrafted rules and parameter selection; limited high-level feature representation	Sensitive to modality differences and acquisition conditions; limited adaptability	Real-time applications requiring efficiency and interpretability
Transform-domain methods	Effective multi-scale and frequency representation; good feature decomposition capability	Performance depends on transformation strategies; possible information loss	Limited robustness across diverse imaging protocols and complex cases	Multi-scale analysis and interpretable fusion tasks
Sparse representation-based methods	Compact feature representation; effective structural information extraction	Require dictionary design and optimization; relatively high computational cost	Limited scalability for large and heterogeneous datasets	Applications requiring interpretable feature representation
CNN-based methods	Strong nonlinear feature extraction; hierarchical feature learning	Require large training datasets; risk of overfitting and dataset bias	Limited generalization across scanners, protocols, and populations	Data-rich scenarios with high fusion performance requirements
GAN-based methods	Improved perceptual quality; effective image enhancement capability	Training instability; potential generation of unrealistic details	Clinical reliability requires further validation	Visual quality enhancement with strict quality control
Transformer-based methods	Global context modeling; effective long-range feature interaction	High computational and memory requirements	Difficult deployment on resource-limited devices; interpretability remains limited	High-performance fusion systems with sufficient resources
Mamba-based methods	Efficient long-range dependency modeling; potential computational advantages	Emerging technique with limited validation	Clinical effectiveness and generalization remain unclear	Future lightweight and scalable MMIF frameworks

**Table 5:** Comparison of Image Fusion Quality Evaluation Metrics.

Ref. and Year	Image Modality	MMIF Technology Based on DL	MI	RMSE	SSIM	CC	EN	AG	SD	SF	ESM	VIF
Rajalingam et al. 2018 [74]	CT/MRI	CNN	1.5160	—	—	—	—	0.0886	—	—	0.7172	—
	CT/MRI		1.7621					0.0822			0.6524	
	MRI/PET		0.8735					0.0844			0.8662	
	MRI/PET		1.5721					0.0893			0.6012	
Zhang et al. 2020 [75]	CT/MRI	IFCNN	0.6144	—	0.4739	—	—	3.4020	—	24.9800	—	0.6799
Ma et al. 2020 [79]	PET/MRI	DDcGAN	—	—	0.9988	0.9012	5.9787	—	0.3519	0.1267	—	0.9792
Xu et al. 2021 [77]	CT/MRI	EMFusion	—	—	0.7780	0.8780	—	-	—	—	—	0.257
	PET/MRI				0.5460	0.9160						0.331
	SPECT/MRI				0.7370	0.9030						0.4600
Fu et al. 2021 [80]	MRI/PET	DSAGAN	—	—	—	—	5.1134	7.6204	—	—	—	—
	MRI/SPECT						5.4984	7.0994				
Mao et al. 2024 [82]	CT/MRI	CFGAN	3.4058	—	0.6836	0.7953	—	—	10.6910	—	—	0.5759
	CT/MRI		3.0756		0.7410	0.8350			9.5857			0.6541
Ma et al. 2025 [84]	CT/MRI	DMFusion	—	—	1.4970	—	4.9090	—	—	—	0.6090	0.5350
	PET/MRI				1.5360		4.3710				0.6720	0.6400
	SPECT/MRI				1.5910		3.9610				0.7600	0.8550
Tang et al. 2022 [86]	MRI-SPECT	MATR	0.9207	—	0.6431	—	—	—	—	—	—	0.3853
	MRI-PET		0.7201		0.6134							0.3081
Ma et al. 2022 [87]	MRI/PET	SwinFusion	—	—	0.9684	—	—	—	—	—	0.7590	—
	CT/MRI				0.9497						0.6529	
Zhao et al. 2024 [88]	MRI/PET	TUFusion	—	0.1721	1.2985	—	—	—	—	—	—	—
	CT/MRI			0.1253	1.4895							
Lin et al. 2024 [78]	CT/MRI	DUSMIF	—	—	0.9614	—	—	7.9619	—	0.1385	—	0.7070
	PET/MRI				0.9500			11.0652		0.1386		0.7825
Liu et al. 2025 [109]	CT/MRI	SAFFusion					4.4526	8.0111	88.2038	34.5237	0.5443	0.4839
	PET/MRI						5.7805	11.5794	80.6647	35.8138	0.8041	0.8014
	SPECT/MRI						5.3198	6.6350	58.1634	20.9895	0.7736	0.8142
Xu et al. 2025 [122]	CT/MRI	MPCT			0.8653	0.8013	5.4295					
	PET/MRI				0.9222	0.8349	5.9991					
	SPECT/MRI				0.9566	0.8900	5.4384					
Liu et al. 2025 [110]	CT/MRI	SSEFusion	2.464		1.4906	0.8196			88.8303		0.5665	0.5502
	PET/MRI		2.2121		1.4790	0.8430			80.7200		0.6787	0.6123
	SPECT/MRI		2.3299		1.5093	0.7667			61.7235		0.7332	0.7716
Xie et al. 2023 [121]	CT/MRI	MRSCFusion	—	—	0.9460	—	—	—	—	7.287	0.6850	0.4630
	PET/MRI				0.9310					8.8150	0.7710	0.5680
	SPECT/MRI				0.9600					6.0150	0.7090	0.6130

## Data Availability

No new data were created or analyzed in this study. Data sharing is not applicable to this article.
